# Single cell epigenetic visualization assay

**DOI:** 10.1093/nar/gkab009

**Published:** 2021-01-28

**Authors:** Sam Kint, Wim Van Criekinge, Linos Vandekerckhove, Winnok H De Vos, Karol Bomsztyk, Diane S Krause, Oleg Denisenko

**Affiliations:** Department of Data Analysis and Mathematical Modeling, Ghent University, Ghent, Belgium; Department of Internal Medicine and Pediatrics, Ghent University, Ghent, Belgium; Department of Data Analysis and Mathematical Modeling, Ghent University, Ghent, Belgium; Department of Internal Medicine and Pediatrics, Ghent University, Ghent, Belgium; Department of Veterinary Sciences, University of Antwerp, Antwerp, Belgium; Department of Medicine, University of Washington, Seattle, WA 98109, USA; Depts. of Laboratory Medicine, Pathology and Cell Biology; Yale Stem Cell Center, Yale University, New Haven, CT 06520, USA; Department of Medicine, University of Washington, Seattle, WA 98109, USA

## Abstract

Characterization of the epigenetic status of individual cells remains a challenge. Current sequencing approaches have limited coverage, and it is difficult to assign an epigenetic status to the transcription state of individual gene alleles in the same cell. To address these limitations, a targeted microscopy-based epigenetic visualization assay (EVA) was developed for detection and quantification of epigenetic marks at genes of interest in single cells. The assay is based on an *in situ* biochemical reaction between an antibody-conjugated alkaline phosphatase bound to the epigenetic mark of interest, and a 5′-phosphorylated fluorophore-labeled DNA oligo tethered to a target gene by gene-specific oligonucleotides. When the epigenetic mark is present at the gene, phosphate group removal by the phosphatase protects the oligo from λ-exonuclease activity providing a quantitative fluorescent readout. We applied EVA to measure 5-methylcytosine (5mC) and H3K9Ac levels at different genes and the HIV-1 provirus in human cell lines. To link epigenetic marks to gene transcription, EVA was combined with RNA-FISH. Higher 5mC levels at the silenced compared to transcribed *XIST* gene alleles in female somatic cells validated this approach and demonstrated that EVA can be used to relate epigenetic marks to the transcription status of individual gene alleles.

## Graphical Abstract

**Graphical Abstract:**
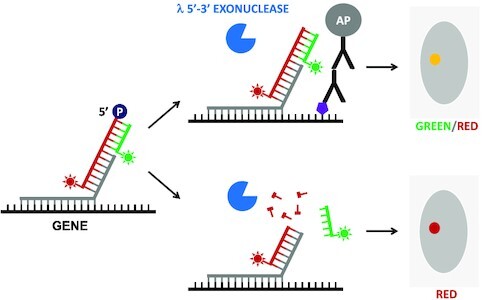
Epigenetic mark visualization at a gene of interest. Alkaline phosphatase (AP) is recruited to the epigenetic mark (purple) as an antibody conjugate. Gene specific oligonucleotides anchor phosphorylated sensor oligo (red) annealed to a detector oligo (green). When the epigenetic mark is present at the gene, the AP-dephosphorylated oligo survives subsequent λ-exonuclease treatment. The presence of the epigenetic mark at the gene is quantitated using the ratio of detector/sensor signal intensities (green/red).

## INTRODUCTION

Epigenetic programs specify cell phenotypes through covalent modifications of histones and DNA, nucleosome position and density, and substitution by histone variants (1–3). While epigenetic alterations drive normal organism development, aberrations in these processes have emerged as hallmarks of cancer and other diseases where particular cell types and individual cells may play critical roles. Conventional approaches used to study epigenetic events, such as chromatin immunoprecipitation (ChIP) and bisulfite sequencing (BS-seq), measure averaged gene epigenetic states in bulk cell populations or tissue fragments and can therefore not be used to estimate the contribution of individual cells to the epigenetic profile of a specimen, let alone combined epigenetic and transcription analysis of gene alleles in a single cell.

To assess epigenetic states in individual cells, sequencing-based and imaging-based techniques have recently been introduced for single cell epigenetic analyses (4). A microscopy-based approach has been developed to measure histone modifications at individual genes in single cells using a proximity ligation assay (ISH-PLA (5)). The advantage of ISH-PLA is that it works *in situ*. However, it involves two enzymatic steps (ligation and rolling circle replication) that yield qualitative rather than quantitative results. Also, the approach only works in a small fraction of cells (<1%). Most of other epigenetic approaches are focused on genome-wide DNA methylation measurements as these have higher sensitivity compared to histone modification analytical tools. DNA methylation is described as the addition of a methyl group at the C-5 position of cytosine (5mC) mostly in CpG dinucleotides. There are 28 million CpGs in the human genome and most of them are methylated in somatic cells (6–8). Locus-specific DNA methylation can be involved in gene silencing, which is an important event in processes such as cell differentiation, X-chromosome inactivation, genomic imprinting, and suppression of repeat sequences (2,9–16). Across the genome, the level of methylation is considered to be inversely correlated with CpG density (17,18). Hypermethylation of dense CpG clusters, called CpG-Islands (CGIs), near promoter regions of mammalian genes is related to transcriptional silencing (9,19–23). CGI methylation status follows a bimodal distribution, where most of islands are either hypo- or hyper-methylated (18). Less studied methylation of sparse intragenic CpGs is most likely involved in the regulation of cryptic intragenic promoters, alternative splicing and cellular differentiation (24–28). Generally, levels of intragenic methylation positively correlate with gene transcription rates.

Low sequencing coverage and resolution remain the key limitations of sequencing-based DNA methylation and histone modification analyses in a single cell (4). Also, these approaches cannot be used to assign epigenomic sequencing data to individual gene alleles in a cell, and more importantly, to the gene transcription status. Another caveat is their limited applicability to the analysis of rare genomic/epigenomic events, such as HIV-1-infected cells present at frequencies 10^−4^ or lower in infected patients. As a consequence, very large numbers of cells need to be sequenced to allow detection of rare events. To enable simultaneous analysis of epigenetic and transcription states of genes of interest in single cells, we developed a novel Epigenetic Visualization Assay (EVA). This method is based on an *in situ* proximity reaction that generates fluorescent signal proportional to the density of an epigenetic mark as DNA methylation or histone modifications at the gene of interest. EVA was tested and validated to quantitate levels of DNA methylation of several target genes in single cells. Combining EVA with RNA FISH enables the simultaneous analysis of DNA methylation and RNA transcription levels at individual gene alleles. EVA can be also used to analyze histone modifications.

## MATERIALS AND METHODS

### Cell culture

J-Lat clones 8.4 and 9.2 from Dr Verdin laboratory were obtained through the NIH AIDS Reagent Program, Division of AIDS, NIAID, NIH (29). HEK-293 and HUVEC cells were grown in DMEM, 10% FBS; Jurkat and J-Lat cells were grown in RPMI1640, 10% FBS. Serum starvation was performed by incubating cells overnight in RPMI1640 supplemented with 0.1% FBS. Serum starved cells were treated with 12-*O*-Tetradecanoylphorbol-13-acetate (TPA, 50 nM) for indicated periods of time, then chilled on ice, washed once with ice cold PBS, and either fixed with cold methacarn solution, or dissolved in Trizol for RNA/DNA isolation.

### Genomic qPCR and reverse transcription qPCR (RT-qPCR)

RNA was isolated using TRIzol reagent (Invitrogen) according to the standard protocol. After cleaning with DNAse I (1 U/10 μg DNA in 20 μl final volume, 15 min at room temperature (RT)) (D9905K, Epicentre/Lucigen), 1 μg of RNA was reverse transcribed with SuperScript IV Reverse Transcriptase (4 U/μl, # 18080093, ThermoFisher) in a 10 μl reaction, 45 min at 37°C. After reverse transcription, cDNA was diluted 100-fold with TE, boiled for 5 min, chilled on ice and quantified using qPCR with primers targeting specific genes of interest (primer sequences are shown in Supplemental Table S1). PCR was performed in triplicates using an in-house qPCR mix. 2 ng cDNA was added to 2.5 μl of in-house 2× qPCR mix containing SYBR Green and 500 nM forward and reverse primers, in a final volume of 5 μl. Amplification (three steps, 40 cycles), data acquisition and analysis were carried out using the 7900HT Real Time PCR system and SDS Enterprise Database software (Applied Biosystems). Standard dilutions of genomic DNA (for genomic targets) or dilutions of pooled reverse transcription reactions (for cDNA targets) were included in each PCR run. Transcript levels were normalized to both LAMC1 and ribosomal protein RPL32 mRNAs, on which the effect of TPA is minimal.

### Probe design

EVA oligo probes for human genes were designed using sequences obtained from the assembly GRCh38/hg38, UCSC genome browser (https://genome.ucsc.edu/cgi-bin/hgGateway). We filtered out repeats and regions with similarity to other genomic sites and designed 50–96 perfect matching oligo probes (30-mers) with spaces between them of ∼20 bp, so that the probe mix covers a genomic region up to 5 kb. All oligos had the same 3′ common sequence (TAG TTT CAG CTT TCC GCA AC) attached, to be used for signal detection. A list of gene-specific oligos is available upon request. Probes for the HIV target were designed similarly, using a reference HIV-1 genome consensus sequence derived by alignment of all complete HIV genomes available in the database from the Los Alamos National Laboratory website (www.hiv.lanl.gov). Oligonucleotides were synthesized by Eurofins Genomics.

### 5mC EVA

EVA detection/signal amplification oligonucleotides (Supplemental Table S1) were HPLC-purified and dissolved in water at 100 μM. Formamide (deionized) was from EMD Millipore Corp., S4117; Dextran Sulfate was from Amersham Pharmacia Biotech, #17-0340-01.

*Day 1:* hybridization. Cells were placed on ice, collected by centrifugation, washed once with cold PBS (20 mM K-phosphate pH 7.2, 150 mM NaCl), and the pellet was resuspended in 1 ml of cold methacarn fixative (three parts of methanol mixed with one part of glacial acetic acid), stored at –80°C. For specimen preparation, 10 μl of cell suspension in methacarn was dropped on a 22 × 22 mm cover slip and allowed to spread. After air drying, cover slips were incubated at 65°C for 10 min, then cooled to RT. 10 μl of hybridization solution (2× SSC, 50% formamide, 10% dextran sulfate, 1% Tween-20, oligo mix (100 nM each)) was pipetted on a glass slide, cover slips (cells down) were placed on top and sealed with rubber cement. Slides were incubated at 95°C for 3 min, transferred to a humidified Petri dish (10 cm), and incubated at 37°C overnight.

*Day 2:* signal amplification and alkaline phosphatase (AP) treatment. Slides were covered by PBS, rubber cement was removed, and coverslips were transferred to a Petri dish with 10 ml of wash solution (2xSSC, 50% formamide, 0.1% Tween-20), and shaken for 30 min at RT. After washing four times with TBST (10 mM Tris–HCl pH 7.5, 150 mM NaCl, 0.1% Tween-20), coverslips were incubated with 10 ml blocking buffer (5% BSA in TBST) for 30 min at RT. 100 μl of blocking buffer with 1:200 anti-5mC monoclonal antibody (clone 33D3, AMM99021, Aviva Systems Biology) and 150 nM CCB oligo solution was pipetted on a piece of parafilm in a humidified Petri dish, coverslips were placed on the drop (cells down), and incubated for 30 min at RT. After 3 × 5 min washes with TBST, cover slips were incubated with 100 μl of 150 nM DAA/TBST for 30 min, washed with TBST 3 × 5 min, incubated in 100 μl TBST with 1:300 AP-anti-mouse antibody (AP-2000, Vector Labs) and 150 nM CCB/TBST for 30 min, washed with TBST 3 × 5 min, incubated with 100 μl of 150 nM DAA/TBST for 30 min, washed with TBST 3 × 5 min. Then coverslips were incubated with 100 μl of 150 nM RF42/TBST with phosphatase inhibitors (PI, *p*-nitrophenyl phosphate 30 mM, β-glycerophosphate 10 mM, NaF 10 mM, Na_3_VO_4_ 0.1 mM, Na_2_MoO_4_ 0.1 mM) for 30 min, washed with TBST/PI 3 × 5 min, rinsed once with TBST and once with AP buffer (50 mM Tris–HCl pH 8.8, 100 mM NaCl, 2.5 mM MgCl_2_), and incubated in AP buffer overnight at RT in the dark.

*Day 3:* exonuclease treatment. Cover slips were washed with TBST 3 × 5 min, incubated with 100 μl of 150 nM DF for 30 min, washed 3 × 5 min with TBST, rinsed with AP buffer, incubate with λ exonuclease (M0262S, New England Biolabs) (45 μl H_2_O, 5 μl 10× buffer, 10 U exonuclease per coverslip) for 1.5 h at RT, washed 3 × 10 min with TBST, rinsed with Tris–HCl 50 mM pH 7.5 with or without DAPI, mounted in 8 μl Vectashield (H-1000, Vector Labs) on a slide and sealed with colorless nail polish. Slides were stored at 4°C in the dark.

### RNA FISH

10 μl of cell suspension in methacarn were dropped on 22 × 22 mm cover slip and spread. After drying, cover slips were incubated at 65°C for 10 min, then allowed to cool to RT. 10 μl of hybe solution (2× SSC, 50% formamide, 10% dextran sulfate, 1% Tween-20, oligo mix (48 oligos to RNA of interest, 100 nM each)) was dropped on a glass slide, cover slips (cells down) were placed on top and sealed with rubber cement. After air drying, slides were transferred to a humidified Petri dish and incubated at 37°C overnight.

For tyramide signal amplification (TSA), coverslips were washed once for 30 min in hybe washing solution at RT, rinsed four times with TBST, incubated with 100 μl of 150 nM RN1-Bio for 30 min, washed 3 × 5 min with TBST, rinsed once with 4× SSC 0.1% Tween 20, incubated with 100 μl of Avidin-HRP conjugate (A106, Leinco Technologies) in 4× SSCT (1:300 dilution) for 45 min. After three washes in 4× SSCT and one in TBST, coverslips were incubated in 100 μl of 100 mM Na-borate pH 8.5, 0.5 mM H_2_O_2_, 1 μg/ml biotin-xx-tyramide (#92176, Biotium) (0.1 μg/ml for rRNA ETS probe) for 30 min at RT. After TBST washes, coverslips were incubated in 100 μl of streptavidin conjugated to desired fluorophore, washed and mounted in Vectashield with or without DAPI.

### RNA FISH + EVA

To combine RNA FISH with EVA, coverslips after biotin-tyramide TSA reaction were incubated in hybridization washing solution for 1 h at RT, excess of solution was drained on paper towel, coverslips were placed on 10 μl of hybridization solution containing EVA oligo mix on a slide, sealed with rubber cement, and processed according to the EVA protocol as described above. Biotin was visualized using rabbit anti-biotin antibodies (1:300, A150-109A, Bethyl) and goat anti-rabbit Alexa Fluor 405 IgGs (1:300, A31556, Thermo Fisher).

### H3K9Ac EVA

Cells attached to coverslips were fixed with ice-cold 70% ethanol and were stored in the same solution at 4°C before use. Coverslips were consecutively rinsed in 80%, and 100% ethanol, then dried. To suppress endogenous peroxidase activity, coverslips were incubated in 0.3% H_2_O_2_ in methanol for 30 min, then airdried. After rehydration in PBS, coverslips were incubated in blocking solution (3% BSA in TBST) for 30 min, then H3K9Ac rabbit monoclonal antibodies (#9649, Cell Signaling) diluted 1:500 in blocking solution were added for 45 min followed by 3 × 5 min washes in TBST. Goat anti-rabbit HRP-conjugated IgGs (T20925, Invitrogen) were added (1:300 dilution in TBST) for 45 min followed by 3 × 5 min washes in TBST. TSA reaction was performed with biotin-xx-tyramide as described in RNA FISH section. Coverslips were dehydrated in 70%, 80%, 100% ethanol, dried and used in EVA as described above.

### Image analysis

Images were collected using a Zeiss Axiovert 200M microscope with Plan-Neofluar 100×/1.3 oil objective. The images were analyzed in Fiji image analysis freeware (30) using a dedicated image analysis script (LociDetector.ijm, available upon request). The analysis essentially detects individual fluorescent signals (loci) within single nuclei and calculates for each locus the ratio of signal intensities between two channels (markers), one reference or detector channel and a second measurement or sensor channel. First, the smoothened background signal of the reference channel is used to segment the nucleus. Then, loci are segmented in both channels using a Laplacian spot detector. Per locus, a concentric band of 5 pixels (rim) is generated, which is used to correct for local intensity variations in the background signal. Then, the signal intensity is measured for each locus and corresponding rim per channel, and the ratio of the corrected intensities is calculated as follows: G/R = [*I*_Locus_Ch2_ – *I*_Rim_Ch2_]/[*I*_Locus_Ch1_ – *I*_Rim_Ch1_], with Ch1 the detector (reference) channel and Ch2 the sensor channel. Before analysis, images were registered by translation to correct for chromatic shift.

### BrdU staining

To detect replicating cells, 10 μM BrdU (5-bromo-2′-deoxyuridine) was added to the culture medium of serum starved Jurkat cells (0.1% FBS overnight) for 0.5, 4 or 5.5 h (31) (a fraction of BrdU-negative cells that replicated EGR1 locus is expected to decrease with increased incubation time). For the last 5 min of BrdU treatment, serum was added to 10% to induce *EGR1* gene (for subsequent RNA FISH assays). Cells were chilled on ice, washed in cold PBS, fixed in ice-cold methacarn and stored at –80°C. 10 μl of cell suspension in methacarn was pipetted onto a 22 × 22 mm cover slip and spread. After drying, cover slips were incubated at 65°C for 10 min and cooled to RT. RNA FISH was done with EGR1 oligo mix probe as described above using TSA and biotin-xx-tyramide. Cells were incubated in 1 M HCl for 20 min at RT, neutralized in 0.1 M sodium phosphate buffer pH 7.4, washed three times in PBS and stained with UltraAvidin DyLight 594 at 1:500 dilution (A427, Leinco Technologies) and rabbit anti-BrdU antibody at 1:500 dilution (#600-401-C29, Rockland) followed by fluorescein-conjugated goat anti-rabbit IgG (FI-1000, Vector Labs). Specimens were counterstained with DAPI and mounted in antifade solution on slides. The number of EGR1 RNA foci (red) per nucleus was recorded for BrdU-positive (green) and negative cells.

### MeDIP

5mC antibodies (mouse clone 33D3, AMM99021, Aviva Systems Biology) used in MeDIP bind to 5mC in a single stranded DNA, thus, before immuno-precipitation, DNA from cells was sheared to small fragments (∼300 bp) by ultrasound and melted by boiling. 1 μg of DNA purified from cells was diluted to 0.5 ml with TE buffer, treated with ultrasound for 30 s (Bronson sonifier, equipped with a microtip), precipitated with ethanol, washed once with 70% ethanol, dried, and dissolved in 10 μl of TE buffer. Before immunoprecipitation (IP), DNA samples were boiled for 5 min, and chilled on ice. 0.5 μg of DNA was used in one IP reaction. MeDIP was done in 96-well plates as described (32,33). Monoclonal antibodies to 5mC, 0.3 μg per IP reaction, were used. Mock IP was done without added antibodies. qPCR analysis of precipitated DNA was done with gene-specific primers as described above. 10% of the amount of input DNA used in IPs was analyzed in parallel by qPCR to estimate efficiency of IP. PCR reactions were run in triplicate. Standard dilutions of genomic DNA were included in each PCR run. Sequences of primers used are in Supplemental Table S1.

### FACS sorting

J-Lat 8.4 cells were latently infected with an HIV-1 defective pseudovirus encoding GFP (26). Cells were either untreated or treated with TPA for 8hrs, chilled on ice, washed with cold PBS, and kept on ice before sorting. FACS Aria II cell sorter (BD Biosciences) was used to sort GFP+ and GFP− J-Lat 8.4 cells. Singlets were selected by forward versus side scatter profiles.

### Statistical analysis

An estimated minimum sample size to detect 1.5-fold difference between two samples (for standard deviation 0.5, power 0.8, α 0.05) was 16. To measure differential methylation, EVA signal ratios of at least 25 cells per specimen were analyzed as G/R as described above. These ratios were normalized using the average G/R of the negative controls (cells treated without λ-exonuclease). Wilcoxon rank-sum test was used to calculate *P* values.

## RESULTS AND DISCUSSION

### Epigenetic visualization assay

EVA is based on an *in situ* proximity reaction where detector (red) and sensor (green) oligonucleotides are both tethered to a gene of interest via gene-specific (30nt) oligos with common (20nt) 3′ sequence (graphical abstract). The assay takes advantage of the ability of 5′-3′ λ-exonuclease to selectively degrade 5′-phosphorylated strands of double-stranded DNA, as opposed to un-phosphorylated strands or single-stranded DNA. λ-exonuclease activity in the assay is controlled by an alkaline phosphatase (AP) recruited as an antibody conjugate to the epigenetic mark of interest. In absence of AP (no epigenetic mark at the gene of interest), the probe remains phosphorylated at its 5′ end, causing the λ-exonuclease to degrade the 5′ half of the detector oligo up to the nick region, thereby releasing the sensor oligo. This results in a low sensor/detector (green/red in graphical abstract) fluorescent signal ratio (G/R). In the presence of AP (epigenetic mark at the gene of interest), 5′-phosphate is removed, protecting the detector oligo from the λ-exonuclease and thus retaining both signals at the locus of interest. This event is detected as a high sensor/detector signal ratio G/R. By virtue of the internal signal normalization, the assay thus allows for quantitative analysis of epigenetic marker density at selected genomic sites.

**Figure 1.**
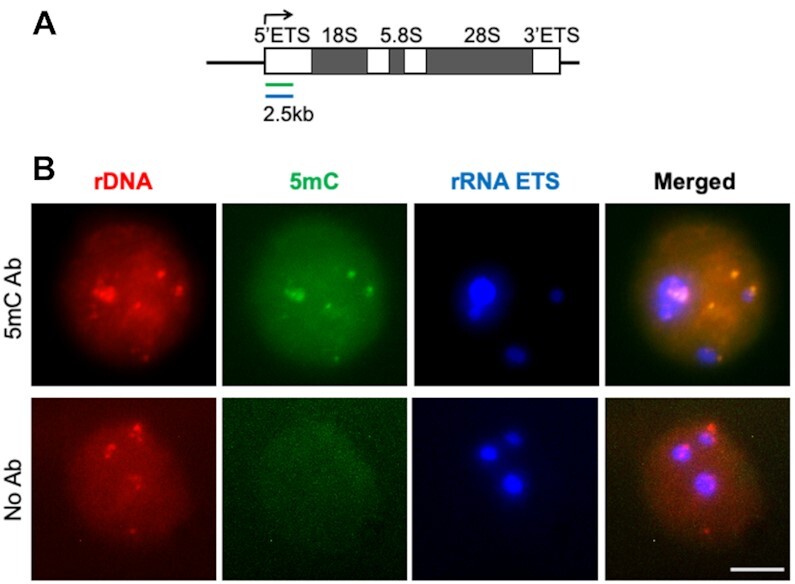
RNA FISH—EVA analysis of rDNA locus. (**A**) rDNA transcription unit and probe design. EVA probe (green) consisted of 50 oligos that cover 2.5kb of the 5′ External Transcribed Spacer (5′ ETS). RNA probe (blue) was to the opposite strand of the same region of 5′ ETS. (**B**) Representative images of rDNA EVA performed in HEK293 cells showing signals in red (rDNA), green (5mC), and blue (rRNA ETS) channels. The upper panel shows RNA-EVA images (5mC Ab), the lower panel shows no-antibody control images (no Ab). Projections of z-stack images of representative cells are shown. Scale bar 5 μm.

To test the design, we began with the detection of DNA methylation (5-methylcytosine, 5mC) at ribosomal DNA loci, which have, due to the multiple copies of rDNA in mammalian genomes, low sensitivity requirements. It has been estimated that about half of rDNA loci are methylated in mammalian cells (34). As a probe we designed a series of 50 oligonucleotides that cover 2.5 kb of 5′ ETS of the human rDNA gene (shown as green line in Figure 1A). This probe was hybridized to fixed human HEK-293 cells overnight. After incubation with 5mC primary and AP-conjugated secondary antibodies followed by washes, detector and sensor oligos were added in the presence of phosphatase inhibitors to block the reaction between the bound AP and detector oligo in solution. Specimens treated without 5mC antibody were used as a control. There are five chromosome arms in human cells that contain rDNA gene clusters (50–80 gene copies per cluster), therefore we expected to see maximum ∼15 foci per cell in HEK-293 cells (hypotriploids with modal chromosome number 64) if they were not clustered. We observed on average ten rDNA foci of diverse sizes per nucleus and all of them also contained the sensor green signal, which reflects the presence of methylated CpGs at the locus (Figure 1B). This was further supported by the absence of detectable green signal at red foci in control cells treated without 5mC antibodies. No difference in green signal intensity was seen between rDNA loci co-localized with 5′ ETS rRNA transcript (blue in Figure 1B) versus those that were not co-localized, suggesting that either the methylation of 5′ ETS region is not related to the gene transcription status, or that active (unmethylated) and silenced (methylated) rDNA repeats are co-clustered in human cells (35).

### XIST EVA

**Figure 2.**
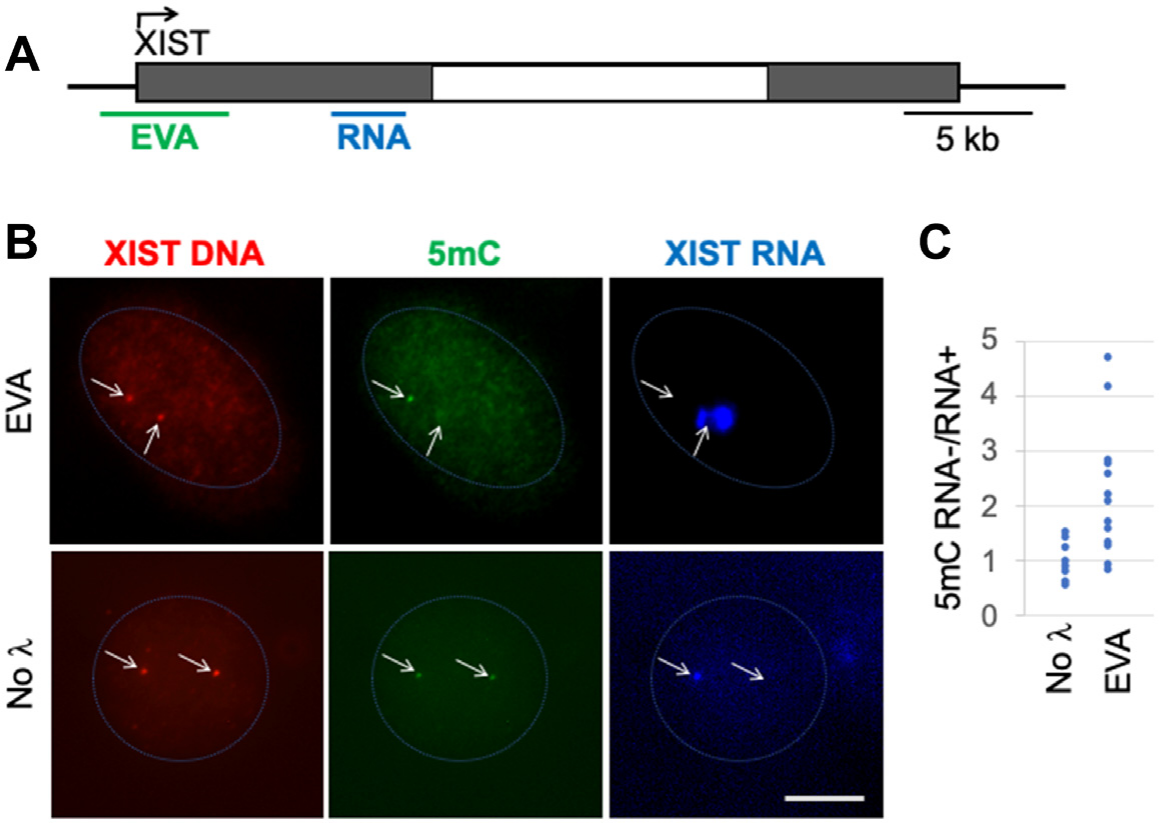
Imprinted XIST gene. (**A**) Human XIST gene locus and probe design. Gray boxes – exons, white box – intron. EVA probe (green) consisted of 85 oligos that cover the ∼4.5 kb which include gene promoter and the beginning of exon 1. RNA probe (blue) was designed to recognize the end of exon 1. (**B**) Representative image of XIST RNA-EVA performed in human female HUVEC. The upper panel shows RNA-EVA images (5mC Ab, EVA), the lower panel shows a no λ exonuclease control (No λ). Arrows point to XIST DNA foci (red). Scale bar, 5 μm. (**C**) Quantitative analysis of images. To measure 5mC density at XIST loci, green and red signals were background corrected, and green-to-red signal intensity ratios were taken for XIST RNA-positive (RNA+) and negative (RNA−) foci. The ratio of 5mC signal at RNA– to RNA+ foci in each cell was calculated. Each dot shown in the graph represents one cell, *P*< 0.05, *n* = 20.

As the methylation status of rDNA copies within and between rDNA clusters is poorly understood, it is difficult to confirm the specificity of the green signal as a readout for 5mC at the rDNA locus by other methods. Therefore, to validate EVA, we subsequently analyzed a gene with known differential distribution of DNA methylation between the silenced and expressed alleles, *XIST*. In human female somatic cells, the *XIST* gene located on the inactive X chromosome (Xi), is transcribed and its promoter is hypo-methylated, whereas its copy on the active X chromosome (Xa) is silenced and hyper-methylated (36,37). Non-coding *XIST* transcript binds to Xi and participates in its inactivation (38,39). Thus, experimentally, Xi can be visualized by *XIST* RNA FISH (38). Therefore, we set out to perform combined *XIST* RNA–5mC EVA assay in human cells. We used an EVA probe to a ∼4.5 kb region including the gene promoter and part of the first exon. The XIST RNA FISH probe consisted of a mix of oligos to the end of exon 1 (both probes are shown in Figure 2A). In test experiments, DNA FISH signal obtained with *XIST* EVA probe in HUVEC cells was very weak in some cells and undetectable in others. To make this signal detectable in the majority (>80%) of cells, we introduced a branched oligo-based signal amplification step (Supplemental file, Figure S1).

Combined XIST RNA-EVA assay in human female cell line HUVEC (Figure 2) showed a 2-fold lower DNA methylation signal at the *XIST* RNA (+) locus compared to the RNA (−) locus (*P* <0.05) (Figure 2B, upper panel, and C). In control cells without λ-exonuclease treatment, green signal was equal at both the RNA-positive and -negative *XIST* foci (Figure 2B, lower panel), and green signal was undetectable in cells treated without 5mC antibody (not shown). These observations provide further support to the interpretation that the green signal quantitatively reflects the density of 5mC at the locus (Figure 2C). Some cells had more than two *XIST* EVA signals per nucleus, most likely due to DNA replication. These cells were excluded from the analysis. We also noted substantial cell-to-cell variation in the 5mC ratio between *XIST* alleles, suggesting a diversity in methylation patterns.

### EGR1 EVA

**Figure 3.**
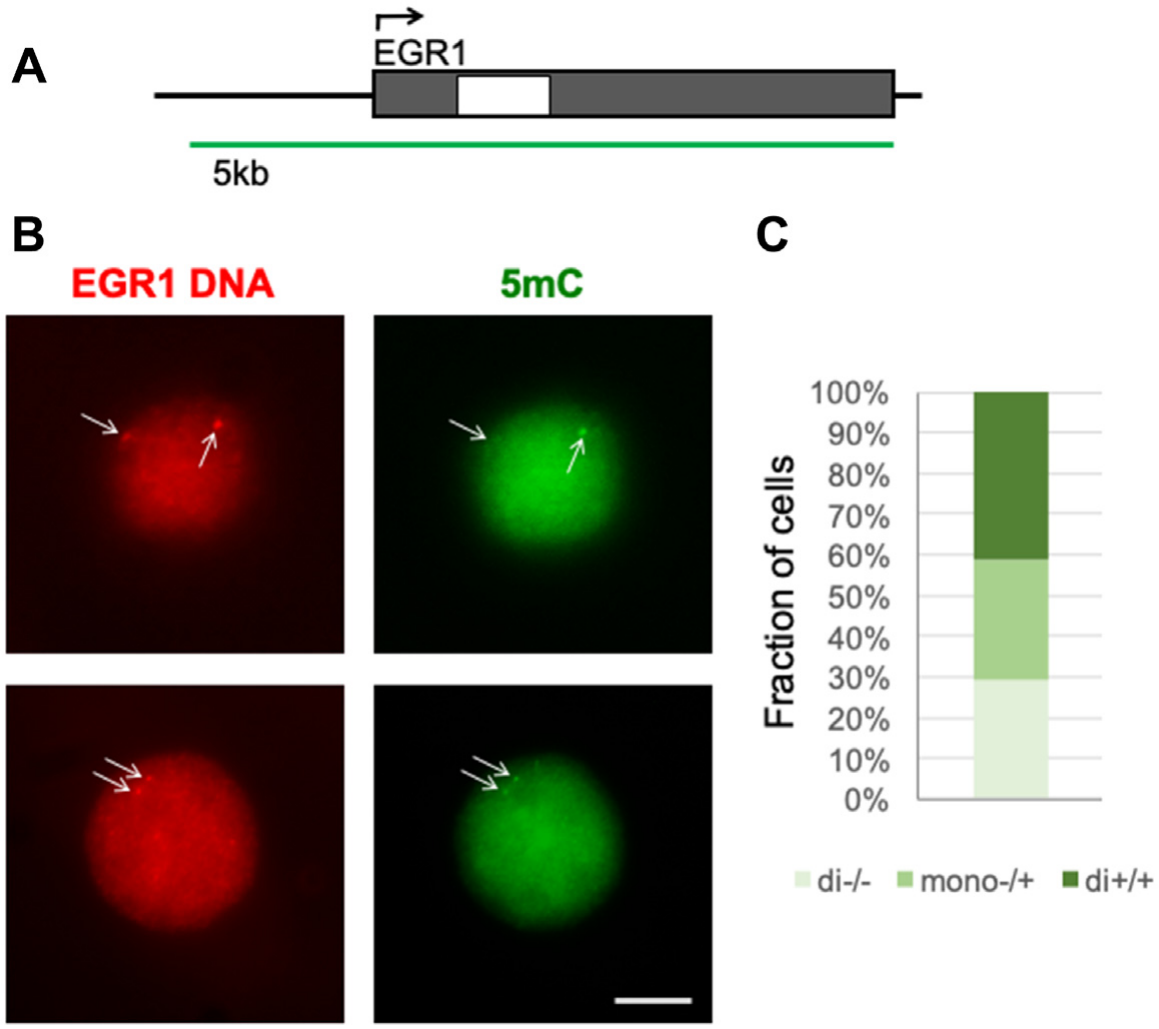
Single copy *EGR1* gene. (**A**) Human *EGR1* gene locus and probe design. Grey boxes are exons, white box is intron. EVA probe (green) consisted of 96 oligos that cover the 5kb including gene promoter and transcribed regions. (**B**) EVA images of *EGR1* locus in Jurkat cells showing DNA methylation in the *EGR1* gene. The upper and lower rows show images of two representative cells. Arrows indicate positions of red foci. Scale bar 5μm. (**C**) Fraction of cells with both EGR1 foci containing green signal (di+/+), one of the foci with green signal (mono+/−), and both foci without green signal (di−/−).

Having confirmed that EVA returns a quantitative readout on methylation status, we next applied EVA to examine *EGR1*, a gene of which the expression can be induced by mitogens. The *EGR1* EVA probe covers gene promoter and transcribed regions (Figure 3A). Without the addition of 5mC antibody no green signal was detected (not shown), while in its presence there was signal (Figure 3B), again suggesting that the ratio of green to red signal (G/R) served as an estimate of the 5mC density per locus. In Jurkat cells, we discriminated three different cell subpopulations, (i) cells with both *EGR1* foci containing green signal, (ii) cells with one of the foci with green signal and (iii) cells with both foci showing no green signal (Figure 3C). These data illustrate that the methylation status of *EGR1* alleles is not uniform in this specific cell population, which may be explained by the dynamic nature of DNA methylation at this locus and by the inefficiency of serum starvation to block cell proliferation in cancer cell lines (40). Treatment of these serum-starved cells with mitogens, such as serum or TPA, rapidly activated EGR1 transcription in the majority of cells, as detected by RT-qPCR (Supplemental Figure S2A) and RNA FISH (Supplemental Figure S2B) (41). Therefore, the RNA FISH-EVA combined assay can be used to examine kinetics of epigenetic changes at the locus during gene activation (Supplemental Figure S3). It should be noted that the EGR1 EVA probe covers both, promoter and gene body regions (Figure 3A), but according to MeDIP analysis (Supplemental Figure S3C), there is little or no methylation around the promoter region (in accordance with the EGR1 methylation map at http://neomorph.salk.edu). Therefore, most of EGR1 EVA 5mC (green) signal likely comes from the 3′ part of the gene body. Shorter EVA probes can be used in a study focused on gene promoters or other regions.

In a fraction of Jurkat cells both EVA and RNA FISH revealed more than two *EGR1* foci per nucleus (3 or 4) (Supplemental Figures S2B and S3B). To test the hypothesis that additional EVA signals were related to DNA replication during the cell cycle rather than unspecific hybridization, we used EGR1 RNA FISH staining combined with 5-bromo-2′-deoxyuridine (BrdU) pulse labeling to detect replicating (S-phase) Jurkat cells. This revealed that BrdU-positive cells (thus representing cells in S or G2 phase) had three or four EGR1 foci, whereas most of the BrdU-negative cells (representing G_1_-phase cells) predominantly had only two foci (Supplemental Figure S2C). These observations support our suggestion that the number of *EGR1* foci reflects the cell's DNA replication status. These data also demonstrate that EVA can be used to monitor the epigenetic status of genes in different phases of the cell cycle.

### HIV provirus EVA

**Figure 4.**
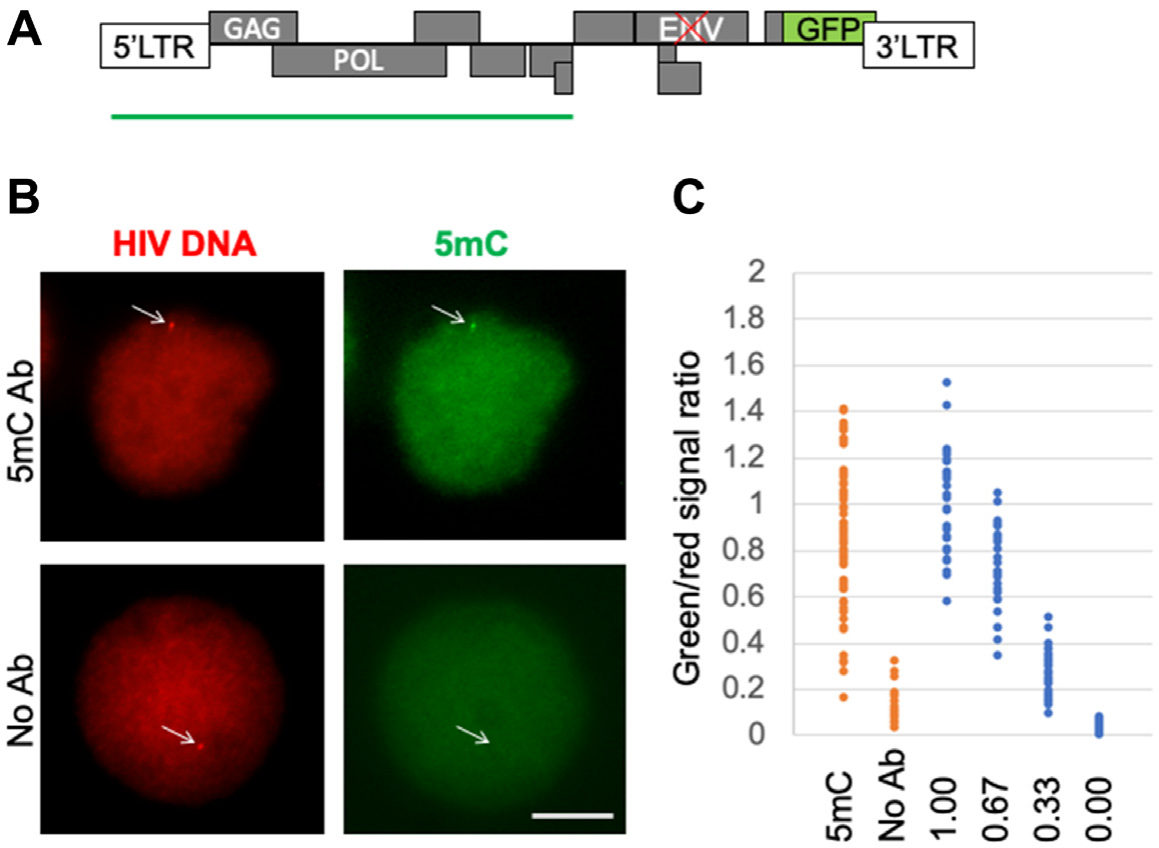
HIV provirus in latently infected cells. (**A**) Schematic overview of the HIV genome and the EVA probe (green bar) that covers 5′ 5kb region of the HIV-1 genome. In the used cell lines (J-Lat), the ENV gene is mutated (red cross), and the NEF gene is replaced with GFP (green box). (**B**) Representative EVA images of the HIV locus in J-Lat 8.4 cell line that contains one copy of the HIV-1 provirus. EVA was done with 5mC antibody (upper row) or without antibody (lower row). Arrows indicate positions of red foci. Scale bar, 5 μm. (**C**) Quantitation of DNA methylation levels of the HIV-1 proviral genome and dynamic range of the HIV EVA signal measurements. Orange: green-to-red EVA signal ratios of HIV locus with (5mC Ab)and without 5mC antibody (no Ab). Blue: HIV EVA was done in the same cells without lambda exonuclease treatment to estimate maximum green signal intensity and technical noise. Different proportions of green oligo were mixed with the same unlabeled oligo (0.00, 0.33, 0.67, 1.00 labeled-to-unlabeled oligo ratios). Each dot represents one cell.

To further explore the utility of EVA, we applied it to the highly relevant topic of rare event detection, the DNA methylation of integrated HIV-1 provirus. In these experiments we used J-Lat 8.4 and J-Lat 9.2 cell lines derived from HIV-1 infected Jurkat cells that were selected to contain one copy of provirus per genome (29). The HIV EVA probe was designed to target the 5′ half of the virus (5 kb, Figure 4A). The HIV RNA FISH probe (48 oligos) was targeting *gag* and *pol* RNA regions. To test if the HIV EVA signal was specific to the HIV-1 locus, we performed a FISH assay with a mixture of two hybridization probes, one probe to the HIV genome (same as used in EVA) and another BAC clone-based DNA FISH probe to the genomic region adjacent to the insertion site (Supplemental Figure S4A). In J-Lat 8.4 cells, HIV-1 is integrated into the FUBP1 gene on chromosome 1, therefore the adjacent BAC clone RP11-156K6 (143 kb) was used. Co-FISH revealed juxtaposition of the HIV probe focus and one of two BAC DNA FISH foci. Additionally, none of the J-Lat 9.2 cells, in which the HIV-1 genome is integrated elsewhere, showed overlap between the HIV probe and the FUBP-1 BAC FISH probe (Supplemental Figure S4B). No HIV genome signal was detected in uninfected Jurkat cells (not shown), all together supporting specificity of the HIV DNA probe used in EVA. This probe produced measurable 5mC EVA signal at the HIV locus (Figure 4B) in the majority of cells (>80%).

To estimate the dynamic range of the assay, we performed an EVA experiment where green detector oligomer was mixed with unlabeled oligomer in different proportions and no λ-exonuclease was used. This approach allowed for estimation of the lowest (0%) and highest (100%) possible G/R values. Average HIV-1 G/R signal intensity measured by EVA is 87% of the highest G/R that could be obtained with this probe (Figure 4C). These results indicate that there is substantial CpG methylation at the HIV locus in J-Lat 8.4 cells, which is consistent with high degree of HIV-1 DNA methylation in this cell line estimated using other approaches (42). Importantly, this type of analysis also allows one to estimate the contribution of technical noise to the cell-to-cell variation in EVA measurements. While the variation in G/R measured in cells that were treated without λ-exonuclease is due to technical noise, in λ-exonuclease treated cells such variation is a result of both technical noise and biological variation. Figure 4C shows indeed that technical signal variation (coefficient of variation, CV = 0.23. CV is defined as SD/mean) is lower than HIV-1 5mC EVA signal variation (CV = 0.35). High 5mC EVA signal variation at HIV-1 locus is in line with the previously described cell-to-cell variation in HIV-1 DNA methylation, detected as a wide diversity of methylation patterns between bisulfite sequencing reads (42). Moreover, it is compatible with the genome-wide cell-to-cell methylation heterogeneity determined by single cell bisulfite sequencing (scBS) approach (43), where CpG methylation concordance between individual cells was estimated as ∼70%, and CV of global methylation measurements between individual cells was 0.40 for a cell culture.

The length of the above-described EVA probes (5 kb) is not suitable for analysis of shorter genomic regions of interest, such as gene promoters or CGIs. To assess lower limits of EVA probe size, HIV probes of decreasing length were tested (Supplemental Figure S5A–C). These experiments define 1 kb as a lower size limit for the current EVA. A 1 kb probe allows detecting differences in 5mC density along the first kb of the HIV locus where the LTR harbors higher density of CpGs, compared to the second kb of the locus (Supplemental Figure S5D). Shorter probe sizes are associated with higher noise in signal measurements. In attempt to reduce the noise, we found that increasing the number of optical slices decreased the variation in the measurements, likely by buffering chromatic shifts in the z-axis between red and green signals (Supplemental Figure S5E).

To assess the ability of EVA to detect quantitative changes in DNA methylation at a locus induced by experimental treatment of cells known to alter 5mC levels, we used J-Lat 8.4 cells exposed to DNA methylation inhibitor DAC (5 uM, 48 h). Such treatment substantially decreased DNA methylation levels at the HIV 5′ LTR region as revealed by MeDIP analysis (Supplemental Figure S6, lower panel). In line with this, EVA revealed a statistically significant decrease in G/R for this locus, demonstrating its quantitative performance.

J-Lat cell lines were selected as cells where integrated HIV-1 was transcriptionally silenced (29). Various agents, including TPA, can activate HIV-1 transcription in these cells (21,42). We used RT-qPCR and RNA FISH to examine HIV-1 transcription in cells treated with TPA. Unlike rapid EGR1 activation (Supplemental Figure S3), no HIV-1 transcription was detected after 30 min of TPA treatment in J-Lat 8.4 cells. However, after 8 hours of treatment, there was robust RT-qPCR HIV-1 RNA signal (>100-fold induction compared to zero time point, data not shown). Yet, RNA-FISH (for HIV-1 transcripts) and FACS (for GFP) revealed that only a small fraction of the cell population (10% and 12.8%, respectfully) expressed HIV locus (Supplemental Figure S7A, B). To link epigenetic changes at the HIV-1 locus to the transcription status of the provirus, we combined EVA with RNA FISH. Results of the combined assay (Supplemental Figure S7A) show that we can detect both, HIV-1 transcript (blue) at the provirus locus and DNA methylation (green) at the same locus in cells that transcribe HIV-1. RNA-FISH tyramide signal amplification (TSA) signal intensity is proportional to the transcript levels at the locus (Supplemental Figure S7C), which enables quantitative analysis of proviral DNA methylation (promoter or gene body) in relation to its transcription status.

### Histone modification EVA

**Figure 5.**
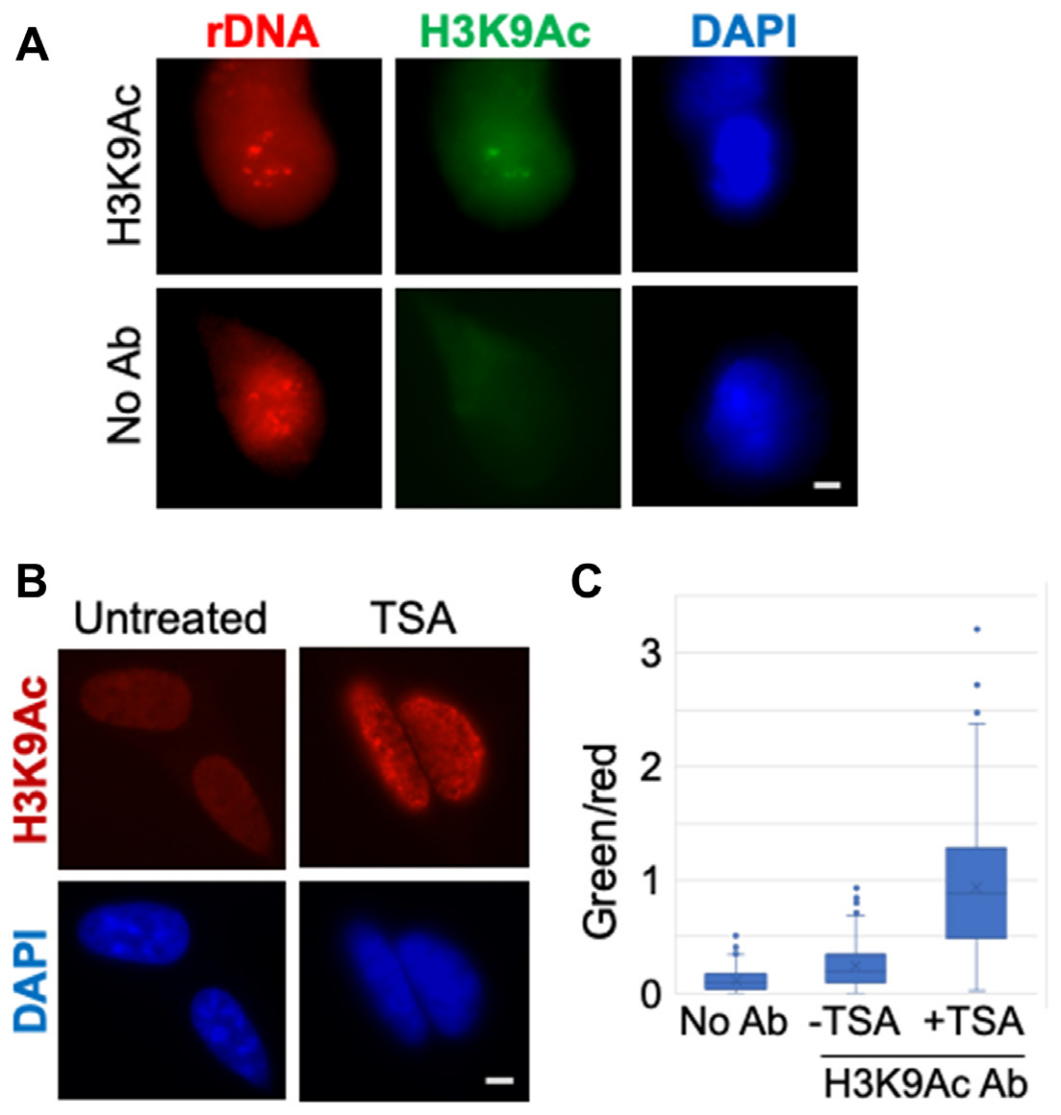
Histone modification H3K9Ac EVA analysis of rDNA. (**A**) H3K9Ac-EVA assay was done in HEK293 cells, grown on coverslips and fixed with cold 70% ethanol, incubated with (top row) or without (bottom row) antibodies. Projections of z-stack images of representative cells are shown. Scale bar, 5 μm. For quantitative measurements, serum-starved cells were treated with or without HDAC inhibitor trichostatin A (1 μM, overnight). (**B**) Immunofluorescent staining, shown are projections of z-stack images of untreated and trichostatin A-treated cells (TSA) stained with H3K9Ac antibodies (red), and counterstained with DAPI (blue). Scale bar, 5 μm. (**C**) results of H3K9Ac-EVA image analysis. Green (H3K9Ac) and red (rDNA) signal intensities were measured, green/red ratios are shown on the graph. *P*< 0.001 for −TSA and +TSA, *n* = 25.

For EVA analysis of histone modifications, simple replacement of 5mC antibodies with those that recognize histones did not work, and several modifications were needed to the initial implementation of EVA. First, methacarn used for cell fixation removes histones from cell nuclei, which is why cells were now fixed in cold 70% ethanol. As shown in Figure 5, antibodies to H3K9Ac stain cell nuclei in such cells, and as expected signal intensity increases in cells treated with the HDAC inhibitor trichostatin A. Second, probe hybridization conditions destroyed histone modification antigens that we tested. Thus, after fixation, cells were incubated with H3K9Ac antibodies followed by secondary antibody HRP-conjugate, then TSA reaction was performed to deposit biotin groups. After this, the regular EVA protocol was used with the rDNA probe and anti-biotin antibody conjugated to AP. EVA analysis revealed differential histone acetylation, high at some rDNA loci clusters and low at others (Figure 5A). Incubation with trichostatin A increased the relative intensity of H3K9Ac EVA signal at rDNA loci (Figure 5B, C, and Supplemental Figure S8). These results demonstrate the potential of EVA approach to study histone modifications.

### Method limitations

In EVA, signal intensity is determined by the probe length as it defines the number of fluorophore groups. For single copy genes, we currently use mixtures of 20–96 gene-specific oligonucleotides. As a result, epigenetic measurements are averaged for the region covered by EVA probe (1–5 kb), and detected changes reflect coordinated behavior of all CpGs present within this region, masking contribution of individual CpGs. Narrowing the EVA probes down to single CpGs will require additional signal amplification, further improvements in hybridization conditions, and more sensitive microscopy techniques.

For a single copy gene, the EVA signal is amplified using branched oligos (Supplemental Figure S1). Such amplification is associated with increased physical distance between the epigenetic marks and the sensor oligos, and therefore decreased resolution. Currently, the signal is amplified 16-fold, and the estimated size of the branched oligonucleotide ‘tree’ is 25 nm, which is comparable to the size of a complex between the primary and secondary antibodies, and on the same scale with 30 nm chromatin fibers. Additional rounds of EVA signal amplification would allow for further decrease of probe size below the current limit of 1 kb, although it may not be practical and would potentially increase influence of other genes forming contact domains (e.g. in topologically associated domains). Size of these complexes also limits the resolution to about 100 bp (considering that DNA is not folded) or more. Therefore, methylation status of individual CpGs present in a short segment of DNA currently cannot be resolved. Instead, EVA estimates synchronous changes in CpG methylation over regions covered by the length of the probe.

EVA is designed for analysis of one epigenetic mark at a time. The same limitation applies to all other available single cell epigenetic technologies (e.g. scBS). Simultaneous analysis of several epigenetic marks (e.g. DNA hydroxymethylation and methylation) by EVA is feasible by using additional enzyme-substrate pairs, and additional colors.

Our observations (Figures 2C and 4C) suggested that there is biological contribution to the 5mC signal variation between cells and between gene alleles in a cell. Although state-of-the-art single cell bisulfite sequencing approaches also reveal high cell-to-cell variance in DNA methylation (43), validation of EVA observations remains a challenge because it will require isolation and bisulfite sequencing of individual gene alleles from the same cells that were examined by EVA, a task which is technically not feasible at present.

## CONCLUSIONS

We have developed EVA, an *in situ* proximity biochemical reaction-based method for quantitative analysis of epigenetic marks at genes of interest in a single cell. This method utilizes inexpensive readily available reagents and standard lab equipment, it is as simple as the conventional FISH assay, it can be multiplexed, and automated for high-throughput purposes.

Despite limitations listed above, EVA has several advantages over sequencing-based methods. First, it allows monitoring methylation status of individual gene alleles in a cell. Second, if combined with RNA-FISH, it provides a means to link changes in gene expression to epigenetic marks in the same cell. Third, if applied to tissues, such as a developing embryo or tumor, EVA measurements can be linked to histological information to advance data interpretation. Finally, it has been demonstrated that this method can also be used to visualize other epigenetic modifications (e.g. histone modifications). The only requirement is a validated antibody.

## Supplementary Material

gkab009_Supplemental_File

## Notes

Present address: Sam Kint, Department of Human Genetics, KU Leuven, Leuven, Belgium.
