# Supplementary material for: Single cell epigenetic visualization assay
Source: Nucleic Acids Res. 2021 Jan 28;49(8):e43. doi: 10.1093/nar/gkab009 (PMC8096246; doi:10.1093/nar/gkab009)
Supplement: gkab009_Supplemental_File [file gkab009_supplemental_file.pdf]

Supplementary data

Single cell epigenetic visualization assay

Sam Kint[1,2], Wim Van Criekinge[1], Linos Vandekerckhove[2], Winnok De Vos[3], Karol Bomzsztyk[4], Diane Krause[5], Oleg Denisenko[4]

[1]Department of Data analysis and mathematical modelling, Ghent University, Ghent, Belgium; [2]Department of Medicine, Ghent University, Ghent, Belgium; [3]Department of Veterinary Sciences, University of Antwerp, Antwerp, Belgium; [4]Department of Medicine, University of Washington, Seattle, WA 98109; [5]Yale University, New Haven, CT 06520

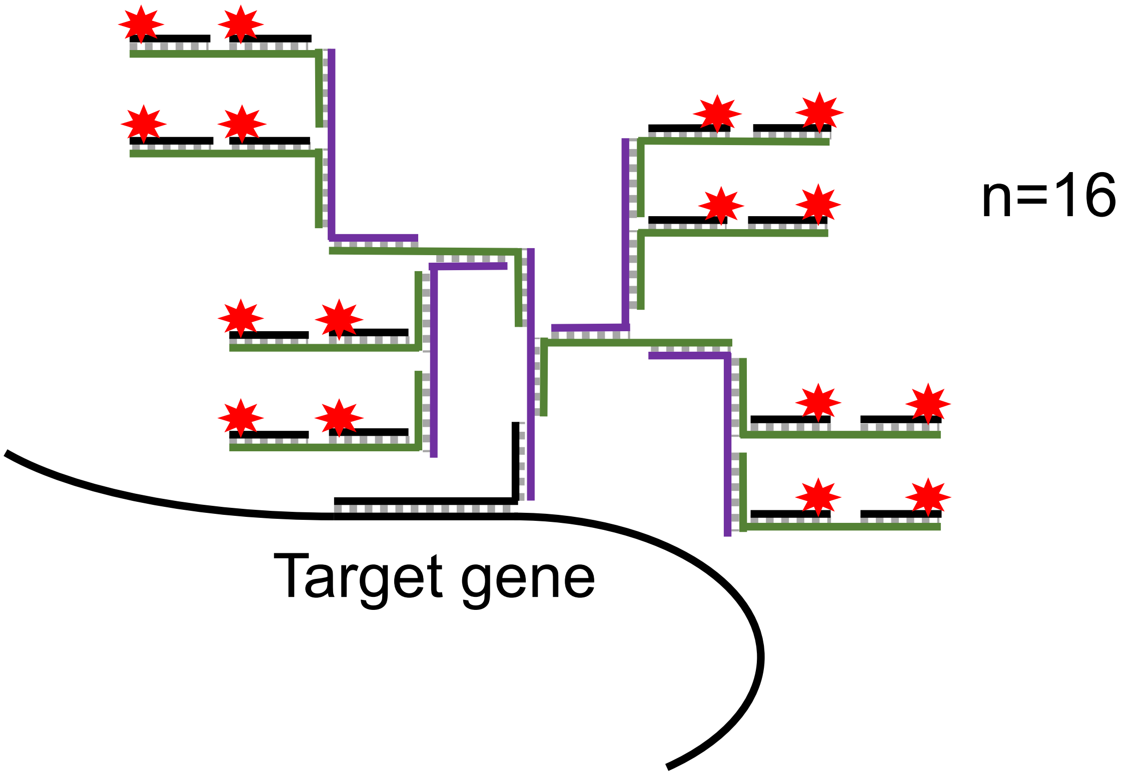

Fig. S1. **EVA signal amplification**. To amplify EVA signal, two oligos (purple and green) that form a tree-like structure were consecutively used. 16x amplification was sufficient to detect a single copy gene in human cells. Estimated size of the 16x “tree” is 25 nm.

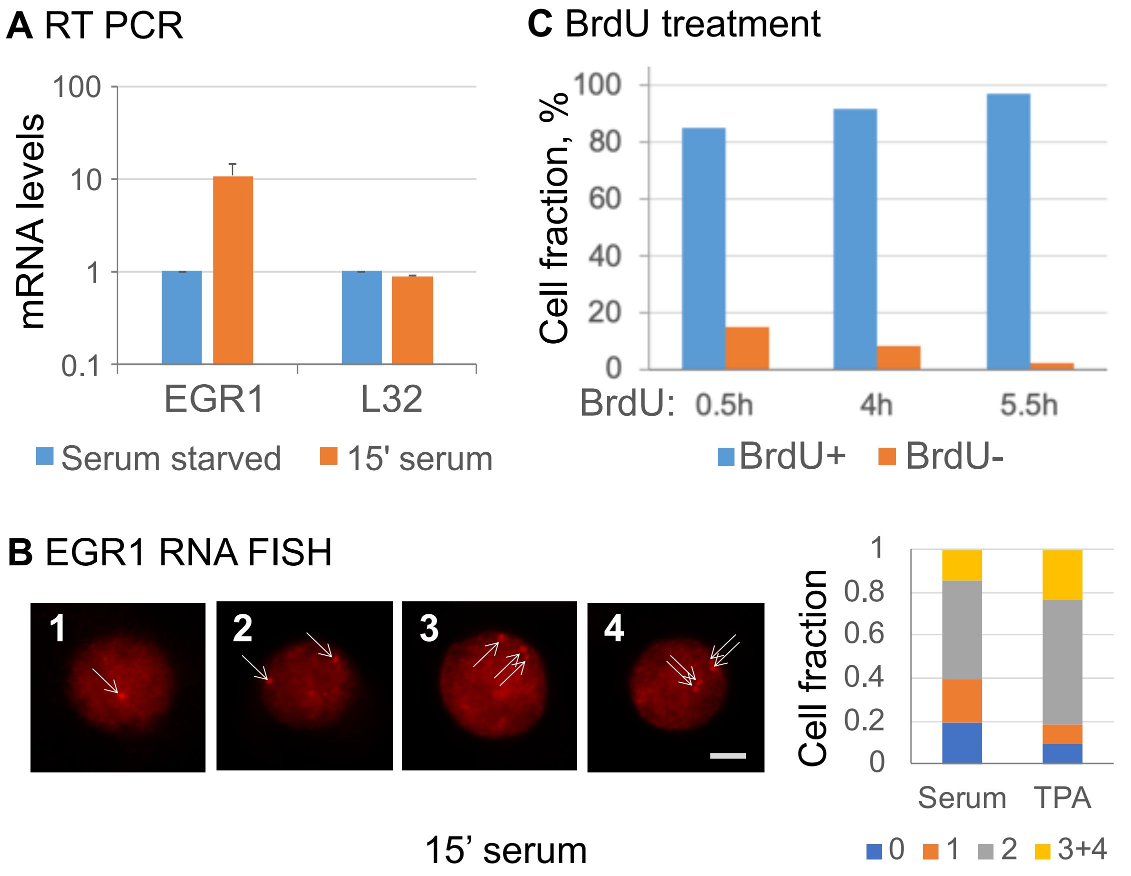

Fig. S2. **EGR1 gene expression in Jurkat cells**. **A**. RT qPCR analysis of EGR1 transcript levels in Jurkat cells either serum starved (blue bars) or treated with serum for 15 minutes (orange bars). Housekeeping gene L32 expression was used as a control. Transcript levels were normalized to β-actin mRNA, fold difference is shown as mean$\pm$SD, n=three independent experiments. **B**. EGR1 RNA FISH analysis reveals bright RNA foci in Jurkat cells treated with serum for 15 min. Representative nuclei images of cells with 1-4 foci per nucleus are shown. Arrows indicate positions of EGR1 transcript foci. Graph shows results of quantitative analysis of cell fractions with 0, 1, 2, 3+4 foci per nucleus in cells treated with either serum for 5 min, or TPA for 5 min; n=5 independent experiments with 25 or more cells analyzed. **C**. EGR1 RNA FISH analysis was done in cells treated with BrdU. Jurkat cells were exposed to BrdU for 0.5, 4, or 5.5 hours, then co-stained with anti-BrdU antibody and EGR1 RNA FISH probe. Graph represents fractions of BRDU (+) (blue bars) and BrdU (-) (orange bars) cells that have 3 or 4 EGR1 foci. Results of one experiment are shown.

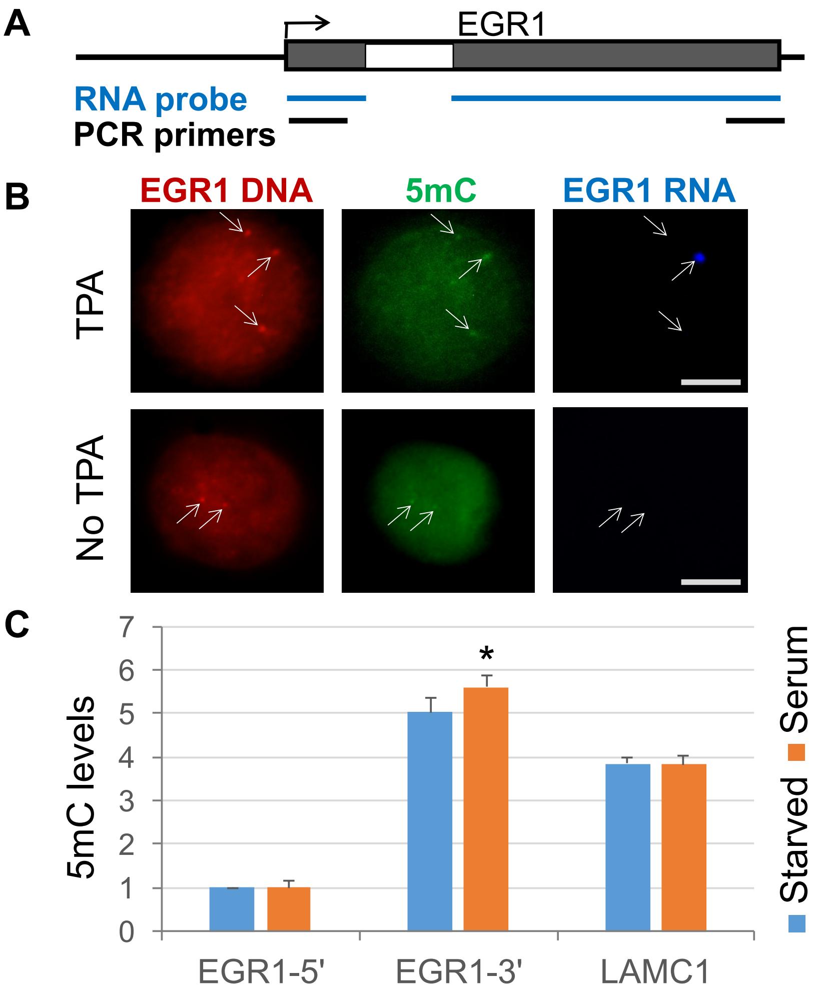

Fig. S3. **EGR1 EVA combined with RNA FISH in Jurkat cells treated with TPA**. **A**. EGR1 gene locus and RNA FISH probe design. Grey boxes represent exons, white box is intron. RNA FISH probe (blue) consisted of 48 oligos that cover the 2.5kb that include both exons. PCR primer pair positions are shown below (black). **B**. RNA FISH – EVA image of one Jurkat cell that was serum starved overnight then untreated or treated with TPA for 5 min showing EGR1 locus (*EGR1 DNA*, red, left panel), DNA methylation (*5mC*, green, middle panel,) and RNA transcript (*EGR1 RNA*, blue, right panel) signals. Arrows point at three EGR1 loci detected in this cell. Scale bar 5μm. **C.** MeDIP analysis of EGR1 locus in Jurkat cells that were serum starved overnight then treated with 10% serum for 15 min (orange pars) or not treated (blue bars). DNA was purified from these cells and precipitated with 5mC antibodies. Primers to the 5' end of LAMC1 gene (exon 28) were used as a no change control. Results are shown as a fraction of input DNA normalized to EGR1-5' signal in starved cells. * $p<0.05$, n=3 independent experiments.

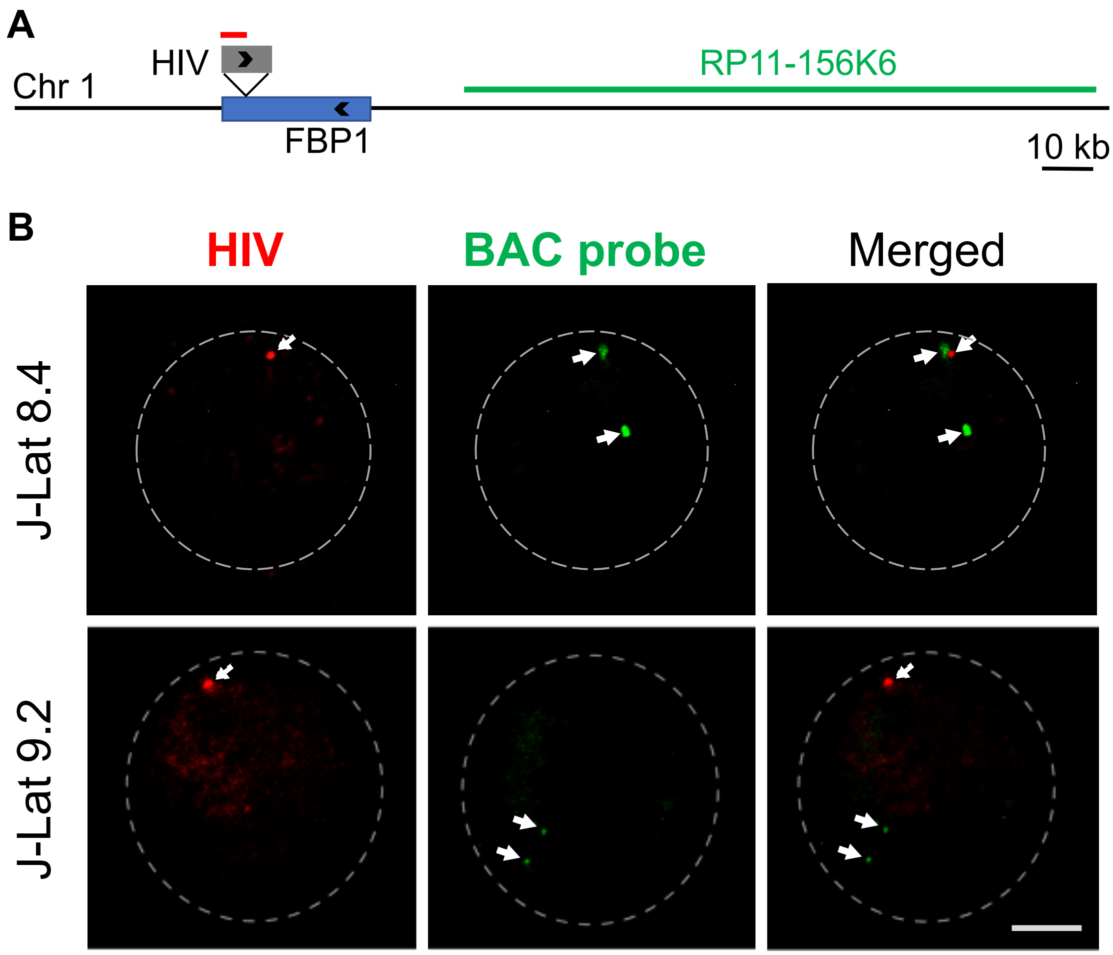

Fig. S4. **Testing HIV EVA probe for specificity**. **A**. FISH probe design. In J-Lat 8.4 cells, HIV-1 is integrated in FUBP1 gene. The HIV EVA probe (red) covers 5’ 5kb region of the HIV-1 genome, and the BAC probe RP11-156K6 (143 kb) (green) covers the genomic region adjacent to the FUBP1 gene. **B**. Representative images of DNA FISH assay done with HIV probe (red) combined with RP11-156K6 BAC probe (green) in J-Lat 8.4 (upper part) and J-Lat 9.2 (lower part) cells. J-Lat 9.2 cell contain the same virus integrated at different genomic site. Cell nuclei are encircled by dashed lines. Scale bar 5µm.

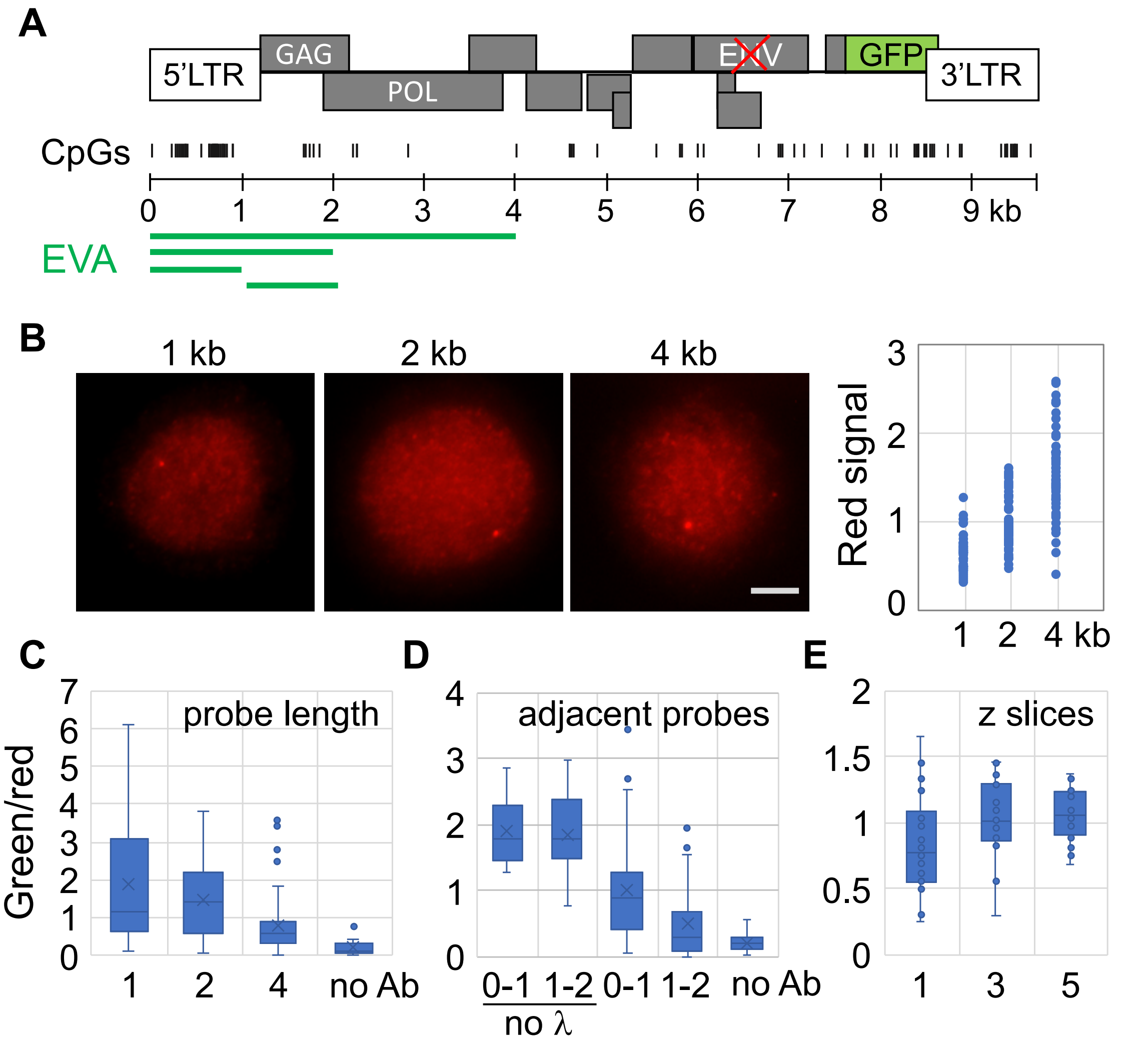

Fig. S5. Estimation of HIV EVA probe length limits. **A**, Schematic overview of the HIV genome, position of CpGs (vertical bars), and three probes that cover 1, 2 and 4 kb regions of the 5' end of the locus (green) that were used in EVA experiments. **B**, DNA FISH was done in J-Lat 8.4 cells with three EVA probes presented above (**A**). Shown are projections of z-stack images of cell nuclei. Scale bar 5μm. Graph represents results of quantitative analysis of signal intensities where each dot corresponds to one cell, n=25. **C-D**, Results of 5mC EVA done in J-Lat 8.4 cells. Ratios of green-to-red signal intensities are shown. No Ab, no antibody control, n=25. **C**, The same 1, 2, and 4 kb probes as in (**B**) were used. **D**, Two adjacent 1 kb long probes that cover 0-1 kb and 1-2 kb regions of HIV locus were used. No λ, no exonuclease was used, No Ab, no antibody control. **E**, 4 kb probe, each focus was imaged with 1, 3, and 5 optical slices (0.5 μm each) along the z axis. In each case, initial focal plane was centered at red focus.

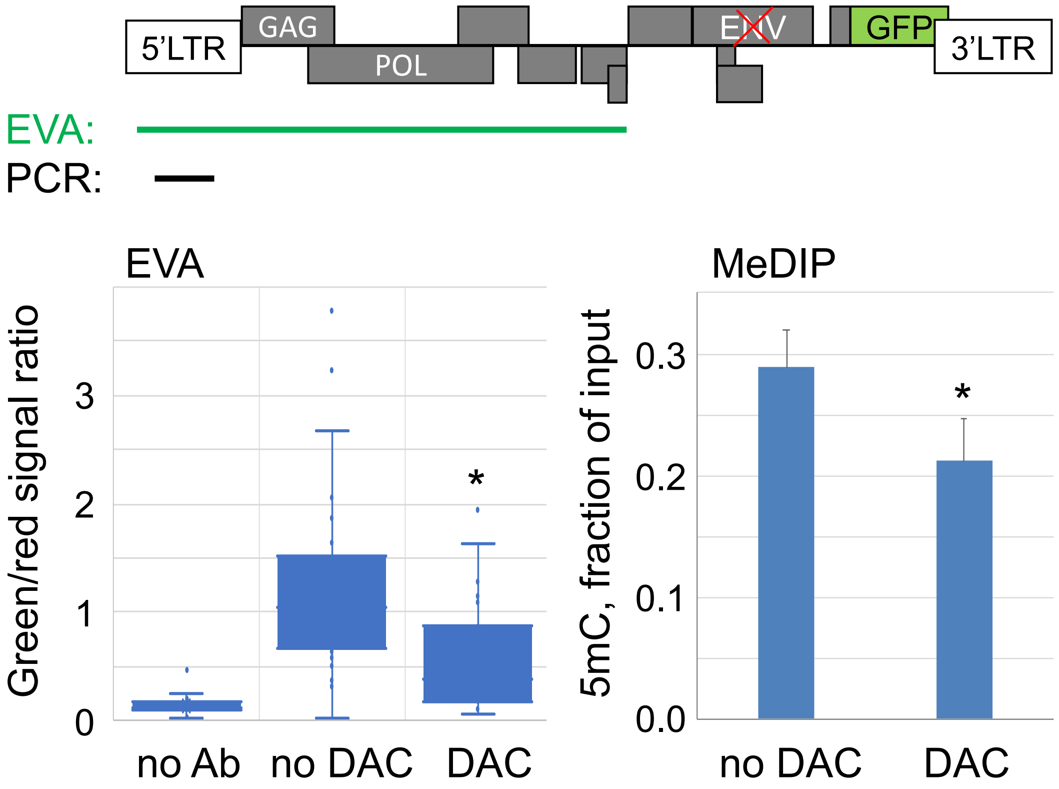

Fig.S6. EVA analysis of DNA methylation changes at HIV locus in cells treated with decitabine (DAC). Upper panel, schematic overview of the HIV genome, position of EVA probe (green) and PCR primers (black bar) used in MeDIP are shown. *Lower panel*, Serum starved J-Lat 8.4 cells were treated or not treated DAC (5 μM, 48 hrs). Cells were either fixed with methacarn and used in 5mC EVA (left graph, n=25) or DNA was purified and analyzed by MeDIP with 5mC antibody and primers to the 5' LTR (n=3 independent experiments of at least 25 cells per experiment), * $p<0.05$.

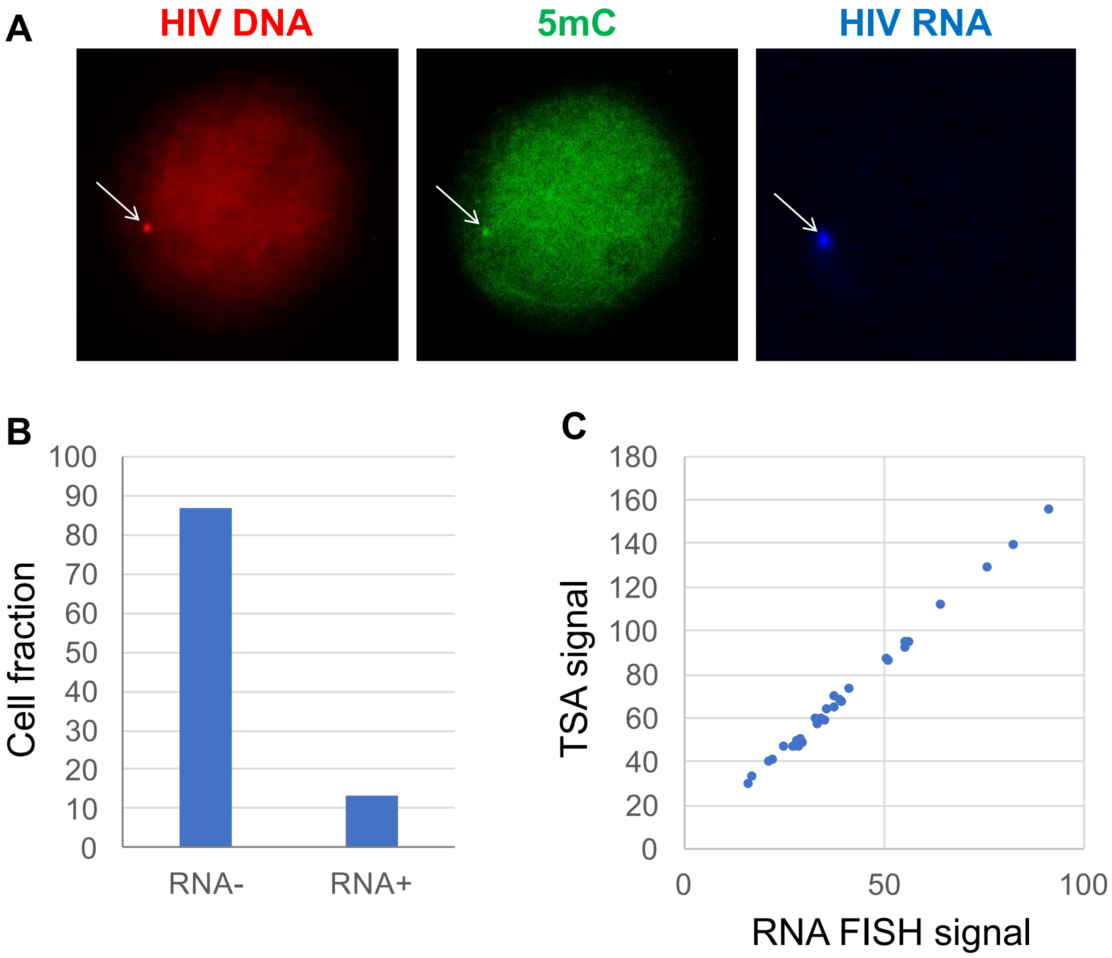

Fig. S7. **Combined HIV EVA-RNA FISH assay**. **A**. RNA FISH – EVA image of a representative J-Lat 8.4 cell treated with TPA for 8 hrs showing HIV-1 locus (red, left panel), DNA methylation (green, middle panel), and RNA transcript (blue, right panel) signals. **B**. Graph represents fractions of cells with detectable HIV RNA FISH signal after 8 hrs of TPA treatment. Results of one representative experiment are shown. **C**. HIV RNA FISH was done in TPA-treated cells (8 hrs) with fluorescein- and biotin- double labeled oligo probe, where biotin was used in tyramide signal amplification (TSA) reaction with biotin-xx-tyramide and visualized with Texas Red – streptavidin conjugate. In RNA-positive cells, fluorescein (*RNA FISH signal*) and Texas Red (*TSA signal*) signal intensities were measured for each focus and plotted on the graph. These data show that TSA can be used for quantitative analysis of HIV RNA transcript levels in RNA-EVA experiments.

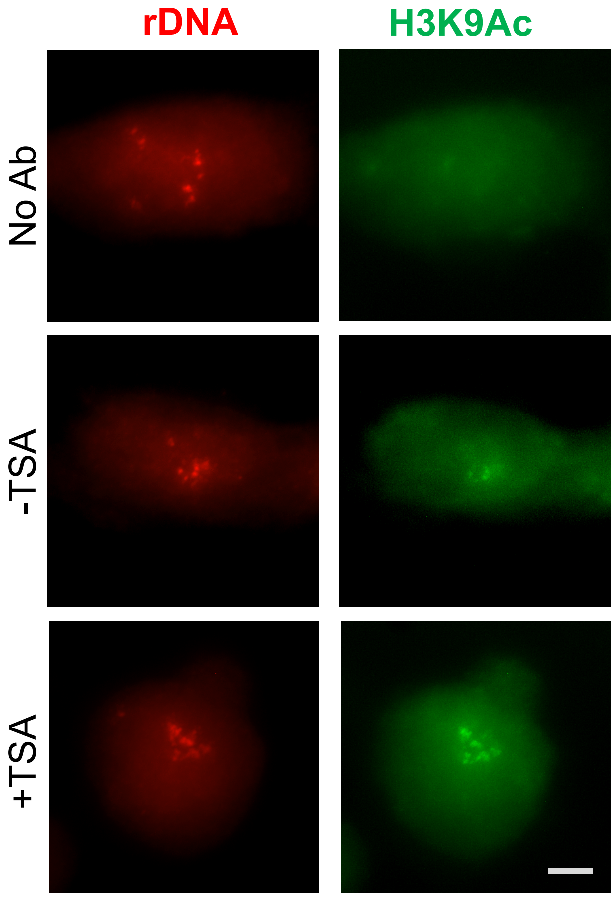

Fig. S8. H3K9Ac EVA analysis of rDNA loci in cells treated with or without histone deacetylase inhibitor Trichostatin A (TSA). HEK293 cells grown on coverslips were either not treated (- TSA) or treated with TSA (+ TSA, 1 μM, overnight), fixed and analyzed by EVA with rDNA probe and H3K9Ac antibodies. *No Ab*, no primary antibodies were added. Representative images are shown. Scale bar 5μm

Table S1. Oligonucleotide sequences

	Name	Location	Sequence
1	hLamc1F	LAMC1 exon 1	CCTTCAACGTGACTGTGGTG
2	hLamc1R	LAMC1 exon 1	GTCGGCCTGGTTGTTGTAGT
3	hL32-F	RPL32 exon 2	AGTTCCTGGTCCACAACGTC
4	hL32-R	RPL32 exon 3	TTGGGGTTGGTGACTCTGAT
5	hEgr1-3'F	EGR1 exon 2	ACTCCTCTGTTCCCCCTGCT
6	hEgr1-3'R	EGR1 exon 2	GTCCTGGGAGAAAAGGTTGCT
7	CCB		TGCTATGGCATGCTTGACAATATGCTATGGCAT GCTTGACAATAGTTGCGGAAAGCTGAAACTA
8	DAA		TTGTCAAGCATGCCATAGCATATAGTTTCAGCTT TCCGCAACTATAGTTTCAGCTTTCCGCAAC
9	RF42		5'-P-TTGTCAAGCATGCCATAGCATAGTTGCGGAA AGCTGAAACTA-3'-TEX615
10	DF		TGCTATGGCATGCTTGACAA-3'-FAM
11	RN1-Bio		TACAGGTGGGATTCGGATTC-3'-Biotin
12	hEgr1-5'F	EGR1 exon 1	CTGCACGCTTCTCAGTGTTC
13	hEgr1-5'R	EGR1 exon 1	AGGCTGGAGAGCTGGTGTC
14	HIV-LTR-F	HIV 5' LTR	TTAAGCCTCAATAAAGCTTGCC
15	HIV-LTR-R	HIV 5' LTR	GTTCGGGCGCCACTGCTAGA
